# Storage in high-barrier pouches increases the sulforaphane concentration in broccoli florets

**DOI:** 10.1371/journal.pone.0192342

**Published:** 2018-02-21

**Authors:** Yoshio Makino, Yuto Nishimura, Seiichi Oshita, Takaharu Mizosoe, Takashi Akihiro

**Affiliations:** 1 Graduate School of Agricultural and Life Sciences, The University of Tokyo, Tokyo, Japan; 2 Department of P-plus Project, Sumitomo Bakelite Co. Ltd., Tokyo, Japan; 3 Faculty of Life and Environmental Science, Shimane University, Matsue, Shimane, Japan; Agriculture and Agri-Food Canada, CANADA

## Abstract

Sulforaphane is a phytochemical that is usually found in cruciferous vegetables and is known to have a depressive effect on gastric cancer. Preliminary investigations showed that the sulforaphane concentration in broccoli (*Brassica oleracea* var. *italica*) florets increased under anoxia. Therefore, in the present study, we examined the effect of different atmospheric conditions on the sulforaphane concentration in broccoli and also tested whether there are concurrent effects on the concentration of ethanol, which is an unfavorable byproduct of fermentation. The sulforaphane concentration in broccoli florets was significantly elevated by 1.9- to 2.8-fold after 2 d of storage under hypoxia at ca. 0% O_2_ and ca. 24% CO_2_ at 20°C, whereas no such increase was observed following storage under normoxia at ca. 0% O_2_ without CO_2_ at 20°C. Furthermore, after 2 d, the sulforaphane concentration under hypoxia was 1.6- to 2.3-fold higher than that under normoxia. These results suggest that storage under hypoxia with high CO_2_ levels can elevate the sulforaphane concentration in broccoli florets. However, the elevated sulforaphane concentration could not be maintained beyond 2 d. There was no significant difference in the concentration of ethanol between florets that were stored under hypoxia with/without CO_2_ or normoxia at 2 d. However, the ethanol concentrations inside the pouches significantly increased between 2 d and 7 d. These findings indicate that the quality of broccoli florets can be improved through storage under hypoxia with high CO_2_ levels at 20°C for 2 d.

## Introduction

Sulforaphane is a phytochemical that is frequently found in cruciferous vegetables and which has been reported to be effective at reducing the risk of gastric cancer. Not only has it been shown that sulforaphane increases the production of a phase II enzyme against this type of cancer [1], but it has also been reported that it depresses the growth of *Helicobacter pylori* (Marshall et al. 1985) Goodwin et al. 1989, which is known to cause gastric cancer [2]. Clarke et al. [3] reported that cruciferous vegetable intake may also lower the overall cancer risk, including the risk of developing colon and prostate cancer, and several recent studies have reported multiple additional mechanisms that are involved in the response to sulforaphane, including the inhibition of cytochrome P450 enzymes, the induction of apoptosis and cell cycle arrest, and the anti-inflammatory effect [4]. In addition, Choi et al. [5] reported that sulforaphane may have antiobesity activity by inhibiting adipogenesis through the down-regulation of peroxisome proliferator-activated receptor γ and CCAAT/enhancer-binding protein α, and by suppressing lipogenesis through activation of the AMP-activated protein kinase pathway.

Several attempts have been made to enhance the sulforaphane concentration in cruciferous vegetables through post-harvest processing [6]. Sulforaphane is known to be produced when myrosinase (EC 3.2.3.1) reacts with glucoraphanin, which is a type of glucosinolate [7]. However, it has been reported that the enzyme and substrate do not coexist in the same cell [8], and so some studies have attempted to bring them into contact using physical methods to injure the vegetable tissues. Van Eylen et al. [9] reported that the high-pressure treatment of broccoli (*Brassica oleracea* var. *italica*) heads at 300 MPa for 35 min caused glucoraphanin to be converted into sulforaphane, while Matusheski et al. [10] found that the mild heating of fresh broccoli sprouts or florets to 60°C prior to homogenization accelerated this conversion process. Furthermore, Esaki and Onozaki [11] reported that grating caused isothiocyanates, including sulforaphane, to be produced in radish (*Raphanus sativus* L.).

As part of a separate study, we accidentally found that the sulforaphane concentration in broccoli was approximately three times higher when the florets were sealed within an acrylic chamber at room temperature for a few days rather than being stored under normoxia, suggesting that hypoxia stimulated sulforaphane production. Therefore, since no previous studies have reported an increase in sulforaphane concentration under hypoxia, in the present study we examined whether hypoxia can increase the sulforaphane concentration in broccoli and what effect this has on the concentration of ethanol, which is an unfavorable byproduct of fermentation.

## Materials and methods

### Samples

No specific permissions were required for these locations/activities. The field studies did not involve endangered or protected species.

Three experiments were conducted in the present study. In Experiment #1 and 2 in Table 1, six broccoli (*B*. *oleracea* var. *italica* ‘Ohayo’) heads were used. Immediately upon arrival at the laboratory, 21 florets (15 g each) were sampled from the 6 heads and treated as independent samples, as we have previously found that the sulforaphane concentration in broccoli shows little variation between heads within the same plot (supporting information). Three of these florets were selected as 0-d storage samples while the remaining 18 florets were randomly assigned to one of two treatment groups (Table 2).

**Table 1:** Broccoli heads used in the present study.

Experiment #	Place of harvest	Harvested date (Month/Date/Year)	Temperature during transport	Selected treatment # ^a^
1	Hyogo Prefecture, Japan (latitude 34° 42′N, longitude 134° 58′E)	1/30/2014	Ambient	1 & 2
2	Kagawa Prefecture, Japan (latitude 34° 10′N, longitude 133° 43′E)	6/1/2014	0‒5°C	1 & 2
3	Nagano Prefecture, Japan (latitude 36° 15′N, longitude 138° 28’ E)	10/5/2014	Ambient	1 − 4

^a^ Selected treatments were presented in Table 2.

Samples were harvested 1 d prior to the initiation of the experiments.

**Table 2:** Treatments used in the present study.

Treatment group	Expected atmosphere	O_2_ transmission rate of pouch (mL m^−2^ d^−1^ atm^−1^)	CO_2_ absorber^d^
1	Normoxia	1.2 × 10^6^ ^a^	−
2	Anoxia	8.0 ^b^	−
3	Anoxia without CO_2_	8.0 ^b^	+
4	Anoxia	64.0 ^c^	−

^a^Micro-perforated polypropylene pouch (thickness, 30 μm; P-plus; Sumitomo Bakelite Co. Ltd., Tokyo, Japan).

^b^High-barrier laminated (nylon/polyethylene) pouch (thickness, 118 μm; Lamizip^®^; As One Co., Ltd., Osaka, Japan).

^c^High-barrier laminated (nylon/polyethylene) pouch (thickness, 40 μm; P-plus; Sumitomo Bakelite Co. Ltd., Tokyo, Japan).

^d^Ageless C^®^ (Mitsubishi Gas Chemical Co. Inc., Tokyo, Japan).

In Experiment #3 in Table 1, twelve broccoli (*B*. *oleracea* var. *italica* ‘Ohayo’) heads were used. Immediately upon arrival at the laboratory, 39 florets (15 g each) were sampled from the 12 heads and treated as independent samples. Three of these florets were selected as 0-d storage samples while the remaining 36 florets were randomly assigned to one of four treatment groups (Table 2).

### Storage methods

Each floret was sealed in a separate pouch, the nature of which depended on the treatment group to which it had been assigned (Table 2). All pouches were stored in an incubator at 20°C. The sulforaphane concentration was measured in three pouches from each treatment group after 2 d, 4 d, and 7 d of storage.

### Gas measurements

The O_2_ and CO_2_ concentrations inside each pouch were measured using a gas analyzer (CheckMate 3; Dansensor A/S, Ringsted, Denmark). The ethanol concentration inside each pouch was measured using a gas detector (XP-3160; New Cosmos Electric Co. Ltd., Osaka, Japan).

### Sulforaphane measurements

Immediately after the floret had been removed from the pouch, 1 g of bud was homogenized in 10 mL dichloromethane, which had been dehydrated using anhydrous sodium sulfate. The homogenate was then centrifuged at 12000 × *g* for 20 min at 4°C, and the obtained supernatant was evaporated to dryness. The residue was dissolved in 2 mL acetonitrile and centrifuged for a further 10 min, following which 1 mL of the supernatant was sampled through a syringe filter (pore size, 0.22 μm⊘), stored in a glass vial, and subjected to sulforaphane analysis.

The sulforaphane concentration in the prepared sample was measured using ultra-performance liquid chromatography with a tandem mass spectrometer (XevoTQMS; Waters Co., Milford, MA, USA) using the electrospray ionization method under the following conditions: capillary voltage, 2.93 V; cone voltage, 21 V; desolvation temperature, 650°C; desolvation gas flow, 900 L h^−1^; mode, positive; MS/MS condition, 174 > 114; collision voltage, 10 V; and dwell time, 0.6 s. Separation was achieved using a C18 column (Kinetex 2.6 μm Polar C18 100 Å, LC Column 100 × 2.1 mm; Phenomenex Inc., Torrance, CA, USA) with a 1-μL injection volume and a solvent flow rate of 0.35 ml min^−1^. The mixing ratio of liquid A (water with 0.1% formic acid) to liquid B (acetonitrile with 0.1% formic acid) was as follows: 0–30 s, 90% A + 10% B; 30–210 s, 90%–60% A + 10%–40% B; and 210–2800 s, 60%– 90% A + 40%–10% B.

### Statistical analysis

The mean values were calculated for each treatment group (*n* = 3 replicates per treatment) and the statistical significance of differences among treatments was tested using Tukey’s honest difference test or ANOVA with Fisher’s least significant difference test in JMP^®^ Pro ver. 12.2.0 (SAS Institute Inc., Cary, NC) with a significance level of *p* < 0.05.

## Results and discussion

### Atmosphere inside the pouches

**Fig 1:**
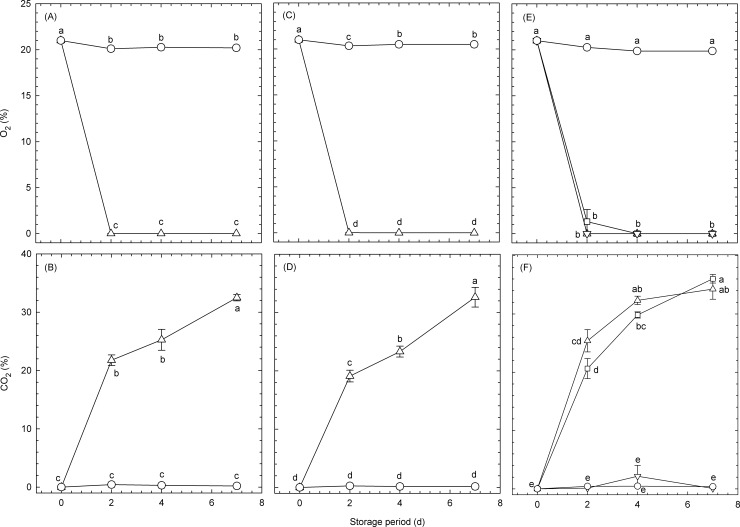
**Temporal changes in [(A), (C), and (E)] O**_**2**_ **and [(B), (D), and (F)] CO**_**2**_ **concentrations inside plastic pouches containing broccoli florets and maintained at 20°C.** Data for (A) and (B), (C) and (D), and (E) and (F) were obtained from Experiment #1, Experiment #2, and Experiment #3, respectively (Table 1). Circles: micro-perforated pouches with O_2_ transmission rates of 1.2 × 10^6^ mL m^−2^ d^−1^ atm^−1^ (normoxia); upward-pointing triangles: high-barrier pouches with O_2_ transmission rates of 8.0 mL m^−2^ d^−1^ atm^−1^ (hypoxia); downward-pointing triangles: high-barrier pouches with O_2_ transmission rates of 8.0 mL m^−2^ d^−1^ atm^−1^ and including CO_2_ absorbers; squares (hypoxia): micro-perforated pouches with O_2_ transmission rates of 64.0 mL m^−2^ d^−1^ atm^−1^ (hypoxia). Values are the means ± SE of three observations from three different biological samples. Symbols followed by the same letter within the same figure indicate that there were no significant differences (*p* < 0.05, Tukey’s honest difference test).

Temporal changes in the atmosphere inside the pouches containing broccoli florets are shown in Fig 1. The O_2_ concentrations inside the high-barrier and micro-perforated pouches with transmission rates of 8 and 64 mL m^−2^ d^−1^ atm^−1^ O_2_, respectively, decreased from 21% to approximately 0% over the first 2 d. By contrast, the CO_2_ concentrations inside the same pouches without CO_2_ absorbers increased from 0.03% to approximately 20% over the first 2 d and showed further increases after 4 d. The CO_2_ concentration inside the high-barrier laminated pouches with CO_2_ absorbers was maintained at approximately 0% during storage, indicating that the use of CO_2_ absorbers is feasible. The O_2_ and CO_2_ concentrations in the micro-perforated pouches with transmission rates of 1.2 × 10^6^ mL m^−2^ d^−1^ atm^−1^ O_2_ were maintained at the same level as in the air. The different atmospheres that were thus achieved were considered suitable for investigating the effects of atmospheric conditions on the sulforaphane concentration in broccoli florets.

### Effect of atmosphere on the sulforaphane concentration in broccoli florets

**Fig 2:**
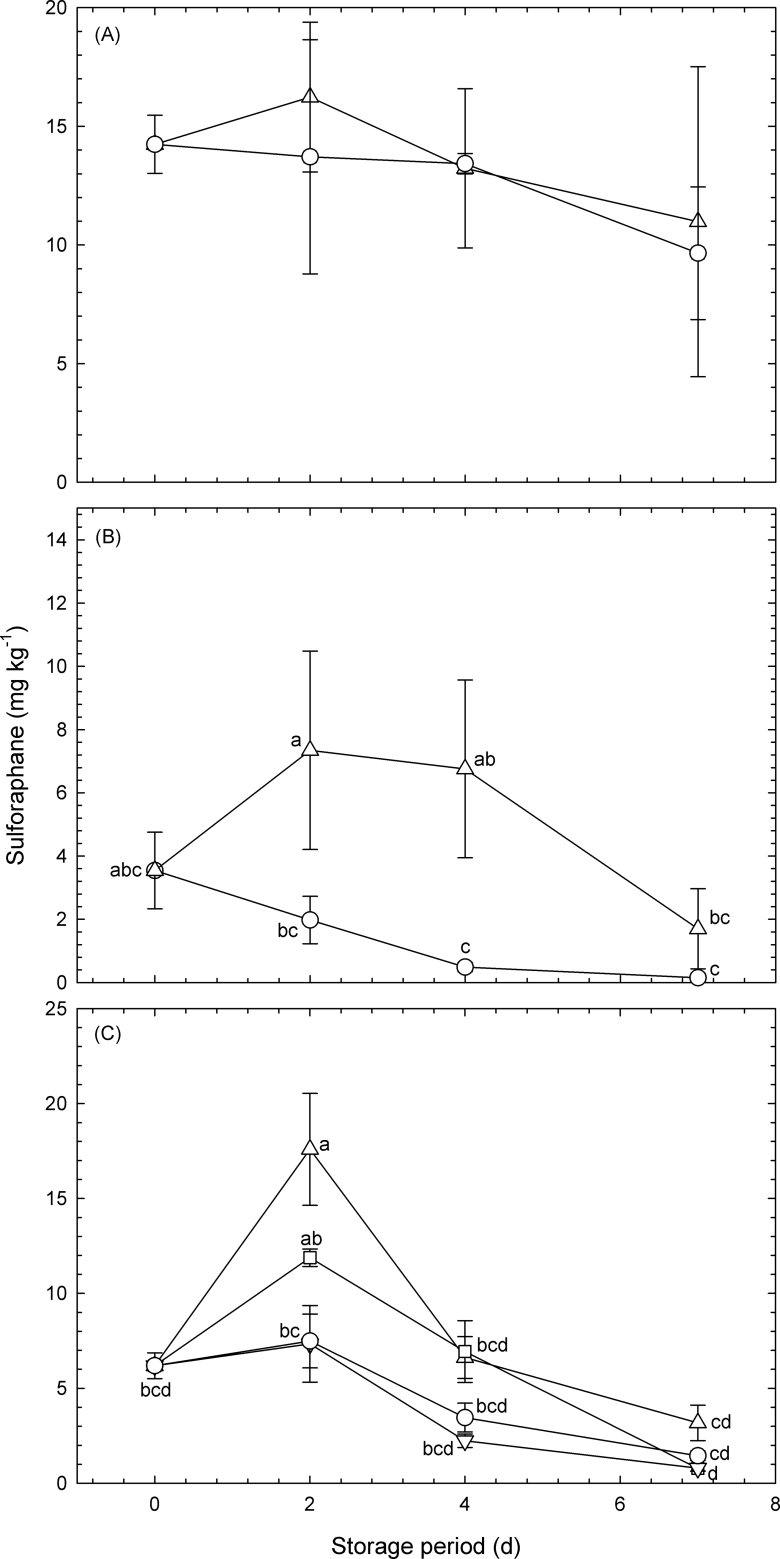
Changes in the sulforaphane concentrations in broccoli florets that had been sealed in plastic pouches for 0–7 d and maintained at 20°C. Data for (A), (B) and (C) were obtained from Experiment #1, Experiment #2, and Experiment #3, respectively (Table 1). Circles: micro-perforated pouches with O_2_ transmission rates of 1.2 × 10^6^ mL m^−2^ d^−1^ atm^−1^ (normoxia); upward-pointing triangles: high-barrier pouches with O_2_ transmission rates of 8.0 mL m^−2^ d^−1^ atm^−1^ (hypoxia); downward-pointing triangles: high-barrier pouches with O_2_ transmission rates of 8.0 mL m^−2^ d^−1^ atm^−1^ and including CO_2_ absorbers (hypoxia); squares: micro-perforated pouches with O_2_ transmission rates of 64.0 mL m^−2^ d^−1^ atm^−1^ (hypoxia). Values are the means ± SE of three observations from three different biological samples. Symbols followed by the same letter within the same figure indicate that there were no significant differences (*p* < 0.05, (B) ANOVA with Fisher’s least significant difference test, (C) Tukey’s honest difference test). The sulforaphane concentration is expressed per unit fresh weight.

Changes in the sulforaphane concentrations in broccoli florets that had been sealed in plastic pouches for between 0 and 7 d are shown in Fig 2. The mean initial value of ca. 6‒14 mg kg^−1^ was similar to that reported previously [12].

In Experiment #1 (Fig 2A), the mean of sulforaphane concentration was higher in broccoli florets that had been sealed in high-barrier pouches and stored for 2 d at 20°C than that in the initial samples. It was also higher than that in micro-perforated pouches (1.2 × 10^6^ mL m^−2^ d^−1^ atm^−1^). After 2 d, the mean value in high-barrier and micro-perforated pouches decreased. These results suggest that the concentration increases on the second day when stored under hypoxia at 20°C. However, there were no statistically significant differences among the mean values.

In Experiment #2 (Fig 2B), as in Experiment #1, the mean of sulforaphane concentration was significantly higher in broccoli florets that had been sealed in high-barrier pouches and stored for 2‒4 d at 20°C than in micro-perforated pouches (1.2 × 10^6^ mL m^−2^ d^−1^ atm^−1^). Thus, the effect of hypoxia on the increase of sulforaphane concentration in broccoli florets was confirmed for the first time. However, the increase in the concentration under hypoxia was reduced to the same level as under normoxia on 7 d.

In Experiment #3 (Fig 2C), the sulforaphane concentration was significantly (2.8-fold) higher in broccoli florets that had been sealed in high-barrier pouches without CO_2_ absorbers and stored for 2 d at 20°C than in the initial samples. The sulforaphane concentration was also 1.9-fold higher in florets that had been sealed in micro-perforated pouches with an O_2_ transmission rate of 64 mL m^−2^ d^−1^ atm^−1^ and stored for 2 d than in the initial samples. There was no significant difference in the sulforaphane concentrations in broccoli florets that had been stored in these two types of pouches.

The sulforaphane concentration was also 2.3- and 1.6-fold higher in florets that had been sealed in high-barrier pouches without CO_2_ absorbers and micro-perforated pouches (high O_2_ transmission, 64 mL m^−2^ d^−1^ atm^−1^), respectively, than in florets that had been sealed in high-barrier pouches with CO_2_ absorbers and micro-perforated pouches (high O_2_ transmission, 1.2 × 10^6^ mL m^−2^ d^−1^ atm^−1^). By contrast, the sulforaphane concentrations in broccoli florets stored in micro-perforated pouches (1.2 × 10^6^ mL m^−2^d^−1^ atm^−1^) and high-barrier pouches with CO_2_ absorbers did not significantly differ from the initial samples. Together, these findings indicate that a rapid reduction in O_2_ and increase in CO_2_ is required to increase the sulforaphane concentration in broccoli florets. Similarly, Makino et al. [13] and Mae et al. [14] previously showed that the γ-aminobutyric acid concentration in tomato (*Solanum lycopersicum* L.) is increased by 148–190% when the fruit are stored for 6–7 d at 25–30°C in an environment of ca. 11% O_2_ and ca. 9% CO_2_ created using micro-perforated pouches rather than under normoxia.

The method proposed in the present study is similar to other pre-distribution treatments that are currently used commercially to improve the taste of fruits, such as the after-ripening of banana (*Musa* spp.) and de-astringency of persimmon (*Diospyros kaki*). Furthermore, sealed packaging is already used to enhance the functional substances in fruits and vegetables and add value to these commodities. Therefore, this method will be put to practical use as a novel pre-distribution treatment in the future.

Interestingly, there was no significant difference in the sulforaphane concentration in broccoli florets stored in sealed pouches at 4 d and 7 d compared with 0 d. This suggests that the increased concentrations that were observed at 2 d may have subsequently decreased and so the atmosphere that is suitable for elevating sulforaphane during the initial 2 d may not be effective in maintaining the concentration for a longer period. Enger et al. [15] similarly reported that in broccoli sprout beverages, sulforaphane is passively absorbed and rapidly conjugates with glutathione via glutathione S-transferases, following which it is sequentially metabolized by γ-glutamyl-transpeptidase, cysteinyl-glycinease, and N-acetyltransferase.

### Effect of atmosphere on ethanol production

**Fig 3:**
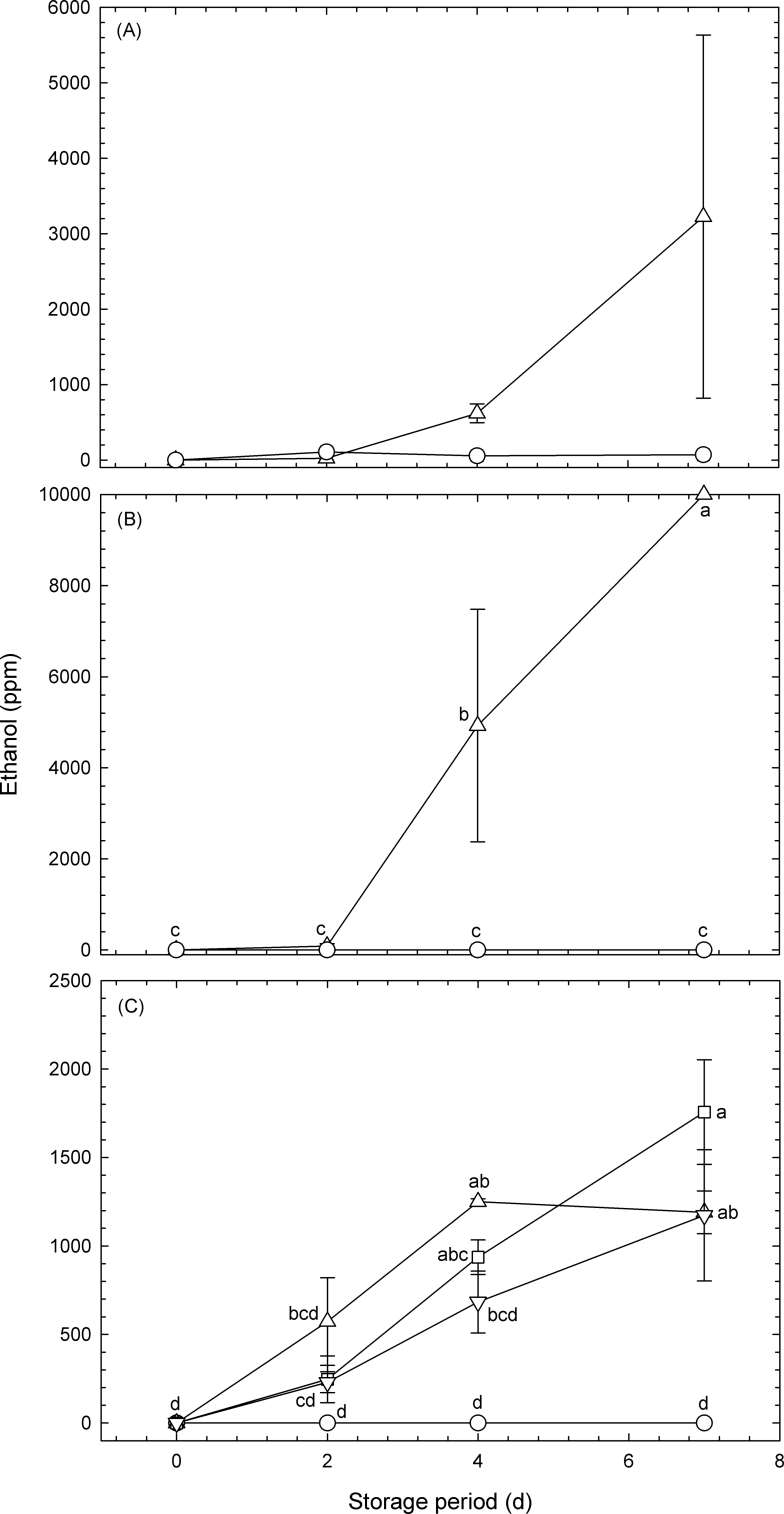
Temporal changes in the ethanol concentrations inside plastic pouches containing broccoli florets and maintained at 20°C. Data for (A), (B) and (C) were obtained from Experiment #1, Experiment #2, and Experiment #3, respectively (Table 1). Circles: micro-perforated pouches with O_2_ transmission rates of 1.2 × 10^6^ mL m^−2^ d^−1^ atm^−1^ (normoxia); upward-pointing triangles: high-barrier pouches with O_2_ transmission rates of 8.0 mL m^−2^ d^−1^ atm^−1^ (hypoxia); downward-pointing triangles: high-barrier pouches with O_2_ transmission rates of 8.0 mL m^−2^ d^−1^ atm^−1^ and including CO_2_ absorbers; squares (hypoxia): micro-perforated pouches with O_2_ transmission rates of 64.0 mL m^−2^ d^−1^ atm^−1^ (hypoxia). Values are means ± SE of three observations from three different biological samples. Symbols followed by the same letter within the same figure indicate that there were no significant differences (*p* < 0.05, Tukey’s honest difference test).

Volatile compounds that result from ethanol fermentation are known to unfavorably affect the flavor of fruits and vegetables [16], making it important that we also consider the effect of the atmosphere on ethanol production. Changes in the ethanol concentration over time as a result of fermentation of the broccoli florets in the pouches are shown in Fig 3.

The ethanol concentration increased over time in the high-barrier pouches with/without CO_2_ absorbers and micro-perforated pouches with O_2_ transmission rates of 8 and 64 mL m^−2^ d^−1^ atm^−1^, respectively. However, there was no significant difference in the ethanol concentration at 2 d between the different pouches, suggesting that broccoli florets that were sealed in pouches with a favorable atmosphere for sulforaphane concentration (i.e., O_2_ transmission rates of 8 and 64 mL m^−2^ d^−1^ atm^−1^ without CO_2_ absorbers) were not exposed to significantly high ethanol concentrations. Ethanol production under hypoxia is known to be a serious cause of off-flavor in horticultural products [16]. However, if the treatment under hypoxia is limited to 2 d, after which it is stored under normoxia, the treatment will not affect the storability of broccoli florets, because there was no significant difference in ethanol concentrations on 2 d between florets stored under normoxia and hypoxia (Fig 3).

## Conclusions

**Fig 4:**
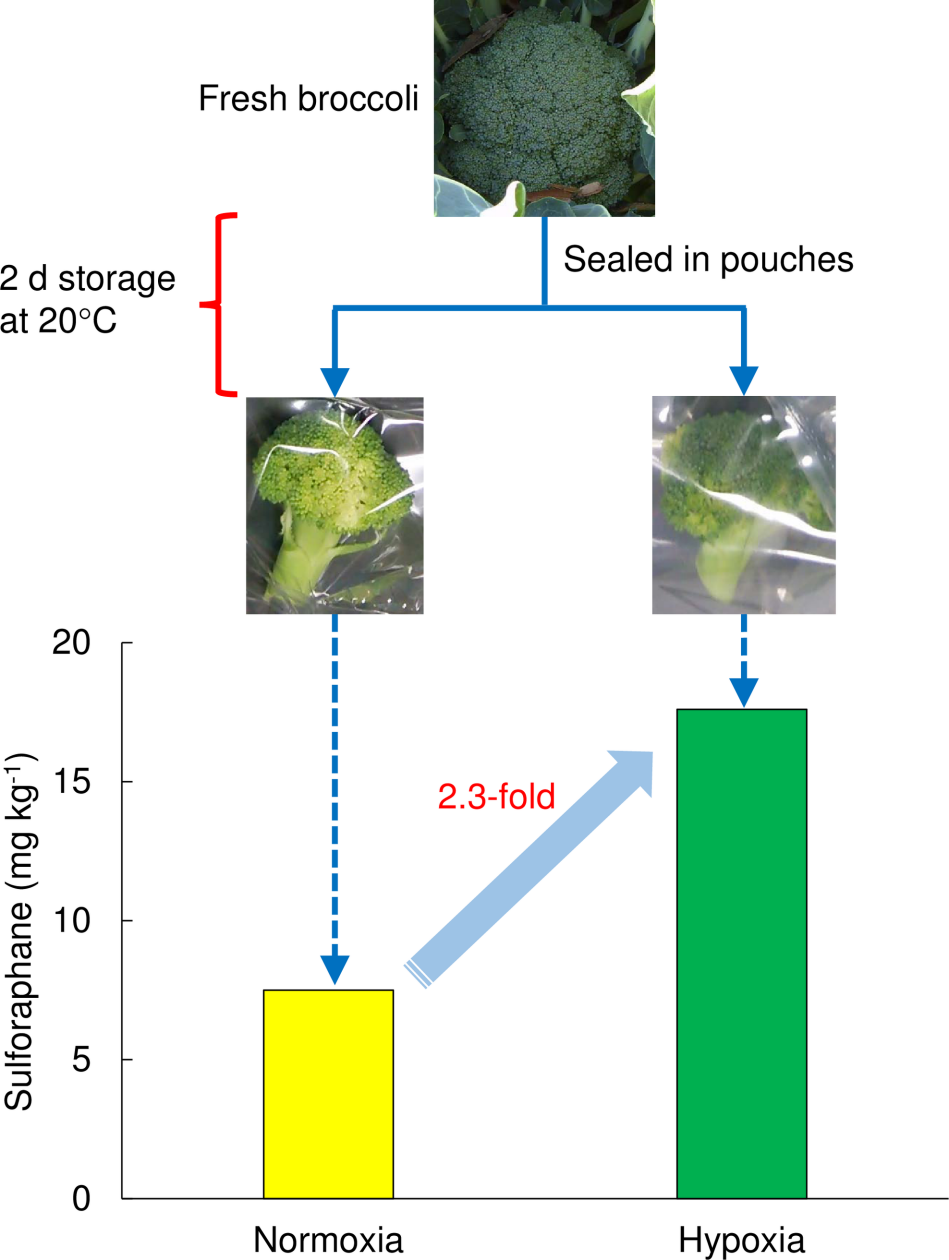
Sulforaphane concentration in broccoli was elevated following storage under hypoxia for 2 d at 20°C.

Storage under hypoxia for 2 d at ca. 0% O_2_ at 20°C was effective in elevating the sulforaphane concentration in broccoli florets (Fig 4). The observed increase in sulforaphane concentration was inhibited when CO_2_ absorbers were also sealed inside the pouches, indicating that a high CO_2_ concentration is also required around the florets.

Interestingly, the high sulforaphane concentration could not be maintained after 2 d, i.e., the increase was transient. Furthermore, although there was no significant difference in the ethanol concentration inside the pouches at 0 d and 2 d, the concentration inside all pouches except the micro-perforated pouches with an O_2_ transmission rate of 1.2 × 10^6^ mL m^−2^ d^−1^ atm^−1^ significantly increased over time. Therefore, the quality of the florets may be reduced or lost when left in the same pouches at the same temperature. It is possible that the refrigeration or cooling of florets sealed in pouches with O_2_ transmission rates of 8 and 64 mL m^−2^ d^−1^ atm^−1^ and without CO_2_ absorbers for 2 d may be effective for maintaining the sulforaphane concentration and flavor of these florets, and so this should be investigated in the future.

## Supporting information

S1 Fig Means and variation of sulforaphane concentrations in fresh broccoli heads. A‒E denote the names of broccoli heads. The heads (Ohayo cultivar) were harvested on January 15, 2014 at a farm in Hyogo Prefecture, Japan (latitude 34°42ʹN, longitude 134°58ʹE). After harvesting, the samples were transported to the laboratory at ambient temperature. The samples were harvested 1 d prior to the commencement of the experiments. Values are the means ± SE of three observations from the same head. No significant difference was found between the means for the heads (*p* < 0.05, Tukey’s honest difference test).
(TIF)
